# Social workers’ determination of when children’s access or potential access to loaded firearms constitutes child neglect

**DOI:** 10.1186/s40621-019-0202-2

**Published:** 2019-05-29

**Authors:** Charles A. Jennissen, Erin M. Evans, Alycia A. Karsjens, Gerene M. Denning

**Affiliations:** 1 Department of Emergency Medicine, Roy J. and Lucille A. Carver College of Medicine, University of Iowa, 200 Hawkins Drive, Iowa City, IA 52242 USA; 2 Department of Social Service, University of Iowa Hospitals and Clinics, Iowa City, IA 52242 USA

**Keywords:** Firearms, Child neglect, Gun storage, Child access prevention laws, Suicide, Social worker

## Abstract

**Background:**

Pediatric firearm-related deaths and injuries are a serious societal problem. Our study’s objective was to determine social workers’ assessment of child neglect with respect to access or potential access to a loaded firearm.

**Methods:**

Study invitations were delivered by e-mail to National Association of Social Workers members designating their practice as “Child/Family Welfare” (N = 4933) in October/November, 2015. Demographics, attitudes regarding child access prevention (CAP) laws, and the ages (4–14 years) at which social workers deemed several scenarios as child neglect were determined. Descriptive (frequencies) and bivariate (chi square) analyses were performed.

**Results:**

485 of 4933 social workers completed the survey (9.8%). Of these, most agreed or strongly agreed (≥92%) there should be laws in place requiring firearms to be stored so unwanted access cannot be obtained by a child, even up to 15 years of age. In a scenario where a child had potential access to a loaded firearm, but never gained access, the presence of a CAP law pertinent to the child in the scenario increased the likelihood respondents would find the situation child neglect for all ages (p < 0.0001 for each age comparison). Moreover, 10.3% felt they could not deem the situation child neglect without the presence of a CAP law, no matter the age of the child. In a scenario where a child gained access to a loaded firearm, the vast majority found this to be child neglect (82–99%, with the percentage varying by the age of the child involved), regardless of the presence or absence of a CAP law and/or an injury being sustained. In addition, when a CAP law was in place, social workers were more likely to find neglect if the child had sustained a firearm-related injury as well (p values ranged from 0.016–0.0081 for age comparisons).

**Conclusions:**

The vast majority of child/family welfare social workers surveyed found it to be child neglect when youth accessed or had potential access to a loaded, unsecured firearm. Results of the study provide support for the passage of universal CAP laws to help protect children equally across states and ensure the safe storage of firearms in homes.

## Background

Firearm-related injury is one of the top three causes of pediatric deaths in the U.S (Dowd & Sege, 2012; National Center for Injury Prevention and Control, 2018), and the country’s youth mortality rate due to firearms is the highest in the world (Centers for Disease Control and Prevention, 1997; Grinshteyn & Hemenway, 2016). Although aggression with firearms remains a big problem, especially among older teens (Srinivasan et al., 2014; Teplin et al., 2014), most firearm-related hospitalizations and many deaths in children are not a result of assault or homicide (Srinivasan et al., 2014; Kalesan et al., 2016a; Kalesan et al., 2016b; Monuteaux et al., 2016; Tseng et al., 2018). Several national studies have found that over three-fifths of hospitalizations due to firearms in children < 16 years of age were not assault-related (Kalesan et al., 2016b; Tseng et al., 2018; Hamilton et al., 2018a).

Suicide is the second leading cause of death in the U.S. for 10–19 year olds (National Center for Injury Prevention and Control, 2016). In fact, the suicide rate for 15–19 year old males increased by nearly one-third from 2007 to 2015, while the rate for females more than doubled (Centers for Disease Control and Injury Prevention, 2017). Firearms play a major role, as 42% of completed teen suicides are executed with firearms (Shenassa et al., 2003). Suicidal ideation in youth can be impulsive, and the urge to commit suicide may be fleeting (Simon et al., 2001; Seiden, 1977). Unfortunately, firearms provide a swift method that is among the most deadly, having a 90% mortality rate (Elnour & Harrison, 2008). Evidence is highly conclusive that firearm access in the home increases the risk of suicide (Grossman et al., 2005; Rivara, 2015; Dahlberg et al., 2004; Miller et al., 2013; Miller et al., 2007; Kung et al., 2005; Hemenway, 2011; Anglemyer et al., 2014; Stroebe, 2013).

Unintentional shootings are a significant cause of firearm-related injuries in children (Srinivasan et al., 2014; Kalesan et al., 2016a; Monuteaux et al., 2016). The majority of firearm-related hospital admissions for youth less than 15 years of age are due to unintentional injuries (Tseng et al., 2018; Hamilton et al., 2018a; Herrin et al., 2018). Approximately one-third of homes in the U.S. with children have one or more firearms (Johnson et al., 2004; Schuster et al., 2000; Hamilton et al., 2018b; Azrael et al., 2018), and they are frequently left loaded and unlocked where they are accessible to children (Schuster et al., 2000; Stennies et al., 1999). More than three-fourths of unintentional shootings occur in the home (Li et al., 1996; Faulkenberry & Schaechter, 2015). In the majority of unintentional firearm-related injuries in children, the individual who pulled the trigger was a minor (Faulkenberry & Schaechter, 2015; Eber et al., 2004; Grossman et al., 1999; Hemenway & Solnick, 2015). One study found that the victim unintentionally shot themselves in one-third of the cases, and approximately half the time they were shot by a friend or member of the family (Li et al., 1996). Securely storing all firearms to prevent their accessibility is associated with a reduction in unintentional firearm deaths in children, even after adjusting for firearm prevalence (Miller et al., 2005).

One approach to modify firearm safety-related attitudes and behaviors would be to apprise parents and legal guardians that improper firearm storage is considered child neglect by society, and could trigger investigation and action by child protective services. A survey of American Academy of Pediatrics (AAP) Section on Child Abuse and Neglect (SOCAN) members examined their attitudes about determining child neglect if an unsecured, loaded firearm was potentially accessed or accessed by children aged 4–14 years old. Even without an injury occurring or a strict child access prevention (CAP) law being present, the majority considered both a child’s potential (range 90–67% for children 4–14 years of age) and actual (range 100–88% for children 4–14 years of age) access to an unsecured, loaded firearm as being child neglect (Evans et al., 2017a). However, it is unclear if social workers, including those who work or have worked as child protective service investigators, would be similar in this assessment.

In order to address this issue, we surveyed members of the National Association of Social Workers (NASW). The survey was constructed to better understand how social workers appraised situations involving children’s potential or actual access to loaded firearms and under what circumstances they would determine the presence of child neglect. In addition, the study evaluated social workers’ attitudes regarding the need for CAP laws for children of different ages.

## Methods

### Survey development and validation

The research team developed the survey tool and performed a pilot test with ten child welfare social workers to validate the survey as previously described (Jennissen et al., 2018). The University of Iowa and NASW Institutional Review Boards approved this study.

### Survey participants

The NASW approved the e-mail distribution of the survey invitation to their members through INFOCUS® Marketing, Inc., which is a firm that provides services to help organizations financially benefit by mail and e-mail marketing of products or services to their members. Survey distribution and data collection occurred from October to December 2015. Potential survey participants were NASW members who stated their practice fell into the category of “Child/Family Welfare.” Study invitees were emailed messages with a link to an online survey available on REDCap, a secure web application for survey research and database management.

At the time of the study, there were 13 states that had more than 200 NASW members whose practice was child/family welfare, and, a randomized sample of 200 child welfare social workers from these states were invited to participate. For states with < 200 NASW members, all members were e-mailed invitations. An e-mail reminder to complete the survey was sent 2-weeks after the first.

A total of 5719 survey invitations were sent with 4933 (86%) deliverable in the first round of e-mails and 4570 (80%) in the second. Of those that had deliverable emails, 1235 (25%) and 1019 (22%) were opened in the first and second round of e-mails, respectively. The marketing agency states that their average open rates for e-mail campaigns are 5–8%. A total of 485 participated in the survey. This was 9.8% of all social workers that were delivered at least one invitation. The response rate considering only those that had opened up at least one e-mail invitation would be appreciably higher and range between 22%–39%, but the exact denominator for this calculation cannot be determined.

Demographic variables for non-respondents could not be obtained from INFOCUS® Marketing. Thus, demographic variables of all who were sent an e-mail survey invitation were utilized to compare against survey respondents.

### Subject-related variables

Study demographic variables included sex, age, race/ethnicity, degree type, and whether the respondent was or had been a parent or child guardian. Subjects were also asked if they were presently or ever had been an investigator of child abuse/neglect for a government agency, lived in a household that owned a firearm, or had personally discharged a firearm. Those that had been a child protection investigator were asked the number of years they served in that capacity, as well as how many firearm-related cases they had investigated and the number of these cases that were eventually classified as founded. Geographic variables were the state (combined into four geographic regions), zip code in which they worked, and the population served (urban, suburban, rural). The rurality of the participant’s workplace was determined using the Rural Urban Commuting Area (RUCA) codes (http://depts.washington.edu/uwruca/ruca-approx.php) and classified as urban, large rural, small rural and isolated rural. For analysis, the three rural categories were combined.

### Attitudes regarding the need for CAP Laws

Respondents were asked to provide a value using a 5-point Likert scale (Strongly Agree, Agree, Neutral, Disagree, Strongly Disagree) to indicate their level of agreement to the following statement, ‘There should be a law requiring firearms to be safely stored (including separately stored ammunition) so that unwanted access to a loaded firearm cannot be gained by a child …’ Three ensuing conditions were provided with the child’s age being ≤11, ≤13, and ≤ 15 years old, respectively.

### Firearms scenarios

Table 1 provides the scenarios used in the survey. Participants were told to ignore their state’s laws and to answer questions based solely on their own judgment. In addition, they were advised that the child in the scenarios was physically and developmentally normal, with no behavioral problems. Finally, subjects were instructed to put a checkmark beside each age for which they believed the scenario constituted child neglect with the selections being 4, 6, 8, 10, 12, and 14 years old.

**Table 1:** Firearms Scenarios from the NASW Child Neglect Survey^a^

Scenario 1: No firearm access by a child	
A man becomes aware that his neighbors store a LOADED firearm in an unlocked drawer where their child could easily gain access to it. He alerts the police. The parents were aware that the firearm was stored in an unlocked drawer.	
1A: Assume NO state laws were violated in this scenario. Is this child neglect if the child never touched the firearm and was 4, 6, 8, 10, 12, or 14 years old.	
1B: Assume the child never touched the firearm, but state law requires firearms to be safely stored so a child of this age cannot gain access. Is this child neglect, if the child was 4, 6, 8, 10, 12, or 14 years old.	
Scenario 2: Firearm access by a child	
A woman notices a child in the yard next door with a firearm. She alerts the police who quickly respond and confirm that the child had a loaded firearm. The parents were inside the house at the time. They were aware that they had stored a loaded firearm in an unlocked drawer.	
2A: Assume NO state laws were violated in this scenario. Is this child neglect, if the child was uninjured and was 4, 6, 8, 10, 12, or 14 years old.	
2B: Assume NO state laws were violated, but the child discharged the weapon causing a serious gunshot wound to the leg. Is this child neglect, if the child was 4, 6, 8, 10, 12, or 14 years old.	
2C: Now assume that the child was NOT injured, but state law requires firearms to be safely stored so a child of this age cannot gain access. Is this child neglect, if the child was 4, 6, 8, 10, 12, or 14 years old.	
2D: Assume the same state law as above was violated, AND the child discharged the weapon causing a serious gunshot wound to the leg. Is this child neglect, if the child was 4, 6, 8, 10, 12, or 14 years old.	

^a^Participants were asked to indicate all ages for which the scenario would, in their professional opinion, constitute child neglect for a child both physically and developmentally normal and with no behavior problems

### Data analysis

SPSS (IBM Statistics Package for the Social Sciences, v22) was used to perform descriptive (frequencies) and bivariate (chi square) analyses. All p values were two-tailed with p < 0.05 defined as being significant.

## Results

### Respondent demographics

Surveys were completed by 485 of 4933 NASW members practicing in Child/Family Welfare who had received the survey invitation by e-mail (9.8%). Demographics of the respondents and comparison with corresponding data for all those having been sent a survey invitation (All Study Invitees) are shown in Table 2. The geographic region with the highest proportion of participants was the South, this proportion was higher than the percentage of NASW members from the South that were sent an e-mail to participate in the study (i.e. study invitees), p = 0.026. Respondents also had a higher proportion from the Midwest.

**Table 2:** Comparison of demographics of survey respondents and all NASW Child/Family Welfare members who were sent an email inviting them to participate in the study^a^

	Study Respondents N = 485 n (col %)^b^	All Study Invitees N = 5719 n (col %)^b^	*P* value
Region			
Midwest	118 (26%)	1341 (23%)	0.026
Northeast	85 (19%)	1214 (21%)	
South	189 (41%)	2083 (36%)	
West	67 (15%)	1081 (19%)	
Sex			
Male	78 (16%)	1044 (20%)	0.09
Female	401 (84%)	4292 (80%)	
Age (years old)			
20-39	109 (23%)	1797 (32%)	<0.0001
40-59	196 (41%)	1801 (32%)	
60 and older	169 (36%)	1965 (35%)	
Ethnicity			
White/Caucasian	362 (79%)	3846 (73%)	<0.0001
Black/African American	72 (16%)	823 (16%)	
Hispanic/Latino	12 (3%)	314 (6%)	
Other^c^	10 (2%)	293 (6%)	
Degree			
BSW	46 (10%)	600 (10%)	0.0006
MSW	376 (78%)	4139 (72%)	
DSW	24 (5%)	191 (3%)	
Other	38 (8%)	789 (14%)	

^a^*Abbreviations: BSW* Bachelors of Social Work, *MSW* Masters of Social Work, *DSW* Doctorate in Social Work

^b^Column total n may not equal N due to missing data

^c^Other ethnicities include Asian, Pacific Islander, American Indian, and Native Alaskan

Females were 80% of survey respondents; a proportion not significantly different from invitees, *p* = 0.09. Around 40% of respondents were 40–59 years of age, a significantly higher proportion than study invitees in this age range, *p* < 0.001. As compared to invitees, survey participants had a higher proportion of White/Caucasians and lower proportions of Hispanic/ Latinos, and other ethnicities, *p* < 0.0001. Seventy-eight percent had a Masters in Social Work, a proportion higher than that of study invitees as a whole, *p* = 0.0006. The survey participants also had a higher proportion of doctorate degrees than study invitees.

Although 93% of respondents reported that their workplace was in an urban area, 41% indicated they primarily served rural populations (Table 3). Almost 70% of participants were parents or guardians. Nearly one-third stated someone in their household owned a firearm. Slightly less than half reported they had personally used a firearm. Forty-three percent indicated they were or had been an investigator, with nearly two-fifths having investigated one or more reported cases of potential child neglect related to a child having accessed a firearm. Of those investigators, 80% had found one or more of the cases investigated to be child neglect.

**Table 3:** Other demographics of NASW Child/Family Welfare survey respondents. N = 485

	n (col %)^a^
Where They Work	
Urban	431 (93%)
Rural	33 (7%)
Population Served	
Urban	146 (32%)
Suburban	126 (27%)
Rural	189 (41%)
Parent/Guardian	
Yes	330 (69%)
No	149 (31%)
Household Owns Firearm	
True	139 (32%)
False	289 (68%)
Have Used a Firearm	
True	202 (47%)
False	226 (53%)
Are/Was an Investigator	
Yes	207 (43%)
No	274 (57%)
Years as Investigator	
Not investigator	274 (57%)
<6	97 (20%)
6 or more	107 (22%)
Number of Cases Investigated^b^	
None	108 (62%)
1-25	57 (33%)
26-100	7 (4%)
>100	3 (2%)
Number of Cases Founded^c^	
None	12 (20%)
1-25	44 (75%)
26-100	2 (3%)
>100	1 (2%)

^a^Column total n may not equal N due to missing data

^b^Number of cases investigated regarding children having access to a firearm by respondents who indicated they are or were an investigator of child abuse/neglect

^c^Number of cases among those investigated regarding children having access to a firearm that were found to constitute child neglect

### Attitudes toward CAP Laws

The vast majority of social workers in the study stated they “Agree” or “Strongly Agree” (≥92%) that there should be state laws requiring the safe storage of firearms in order to prevent children from accessing them, even if the child was 15 years of age. In fact, the percentages who stated they “Strongly Agree” with the need for strict CAP laws were 90% (387/430), 85% (368/434) and 81% (352/435) for children up to 11, 13, and 15 years of age, respectively.

There were some differences that were statistically significant following comparative analysis of reported attitudes. For example, males less commonly agreed that CAP laws were needed covering ages 11 and 13 years. Participants in households with firearms or who had used a firearm also less commonly agreed than their peers. However, the total number who disagreed in every case where there were differences was very small (≤11).

### Results for Scenario 1

**Fig. 1:**
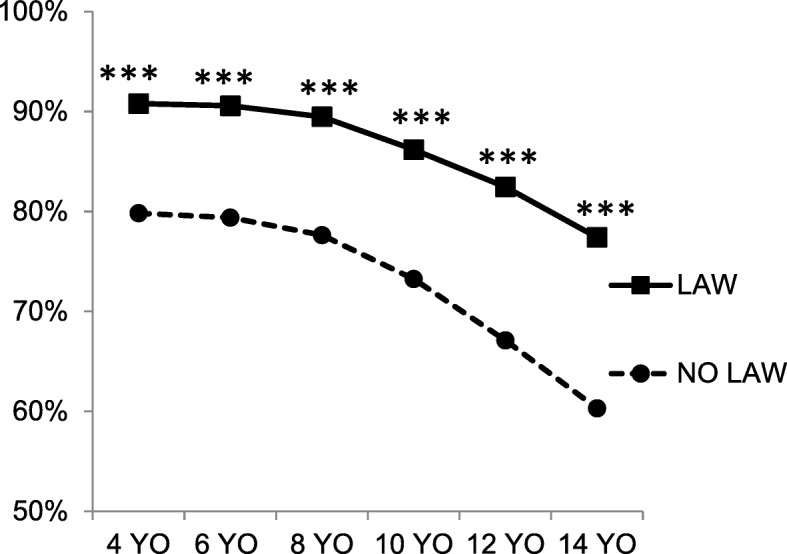
A child has potential access to an unlocked, loaded firearm. The graph compares the proportion of survey respondents who considered Scenario 1 as child neglect for the indicated ages in the absence (NO LAW) or presence (LAW) of a CAP law. Asterisks (*) indicate statistically significant differences for pairwise comparisons: ***, *p* < 0.0001

In Scenario 1, a child had potential, but not actual access to a loaded and unlocked firearm. The majority of social workers who participated in the study found that the scenario constituted child neglect. Figure 1 shows the proportion of respondents who considered the scenario child neglect at the indicated age in the absence (No Law) and presence (Law) of a state CAP law. For each age, a significantly higher proportion of respondents made the determination of child neglect if there was a state law than when there was not, *p* < 0.0001 for all ages. In the absence of a CAP law, 20.2% (92/456) of participants indicated that they would not consider it child neglect for any of the ages listed, including 4 years old. This was true for 9.2% (42/456) of participants when a CAP law was included in the scenario. These participants in each case were a significant contributor to observed differences. When this group was excluded from comparisons, significance was still reached when comparing maximum ages of 12 years old (*p* = 0.006) and 14 years old (*p* = 0.0008) with and without a law.

A comparison was performed between the 92 participants who did not regard Scenario 1A (no strict CAP law) to be child neglect for any of the ages listed, and all other respondents. Observed differences are summarized in Table 4. Relative to other respondents, the group that answered no age was child neglect had a higher proportion of males, of respondents serving rural communities, of persons living in households that owned a firearm, and of persons that had fired a gun. This group also had lower proportions that agreed with the need to have a CAP law covering 11 (84% vs. 99%), 13 (85% vs. 96%), and 15 (80% vs. 94%) year olds, *p* < 0.001 in each case.

**Table 4:** Comparison of Social Workers with Differing Beliefs of Child Neglect Regarding a Child’s Potential Access to a Loaded Firearm When No Cap Law is Present^a^

	No Child Neglect at Any Age n (col %)^b^	Child Neglect for Some Ages n (col %)^b^	*p* value
Group N	92	364	
Sex			
Male	22 (24%)	55 (15%)	0.042
Female	68 (76%)	307 (85%)	
Population Served			
Urban	22 (25%)	117 (34%)	0.037
Suburban	19 (22%)	97 (28%)	
Rural	47 (53%)	133 (38%)	
Household Owns Firearms			
True	36 (44%)	103 (30%)	0.018
False	46 (56%)	241 (70%)	
Have Used a Firearm			
True	53 (65%)	149 (43%)	<0.001
False	28 (35%)	196 (57%)	
“There should be a law requiring firearms be safely stored (including separately stored ammunition) so that unwanted access to a loaded firearm cannot be gained by a …..”			
Child Age ≤11			
Strongly Agree	61 (74%)	324 (94%)	<0.001
Agree	8 (10%)	16 (5%)	
Neutral	6 (7%)	1 (0.3%)	
Disagree	4 (5%)	% (1%)	
Strongly Disagree	3 (4%)	0 (0%)	
Child Age ≤13			
Strongly Agree	53 (65%)	313 (89%)	<0.001
Agree	16 (20%)	26 (7%)	
Neutral	5 (6%)	5 (1%)	
Disagree	4 (5%)	5 (1%)	
Strongly Disagree	4 (5%)	1 (0.3%)	
Child Age ≤15			
Strongly Agree	51 (61%)	299 (85%)	<0.001
Agree	16 (19%)	32 (9%)	
Neutral	8 (10%)	7 (2%)	
Disagree	4 (5%)	11 (3%)	
Strongly Disagree	4 (5%)	1 (0.3%)	

^a^Characteristics of a group who did not regard Scenario 1A (potential child access to a loaded firearm with no strict CAP law) as being child neglect for any of the ages listed in the study, including 4 years of age, as compared to other survey respondents.

^b^Column n total may not equal N due to missing data.

### Results for Scenario 2

**Fig. 2:**
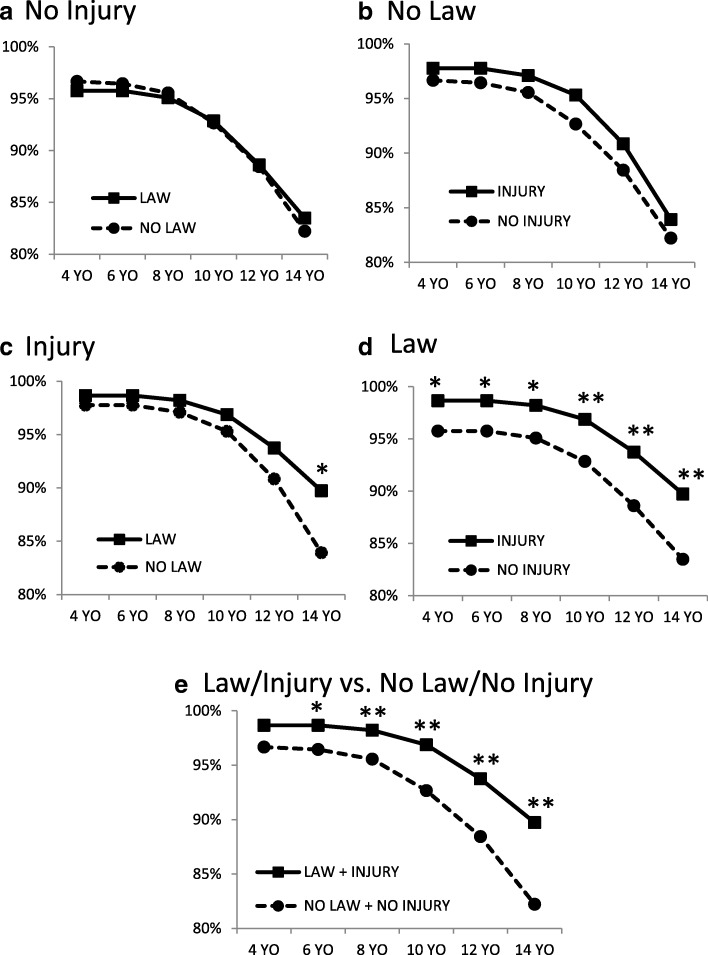
A child has gained access to an unlocked, loaded firearm. The graphs compare the proportion of survey respondents who considered Scenario 2 as child neglect for the indicated ages. **a** scenario where there was no injury to the child and in the absence (NO LAW) or presence (LAW) of a CAP law. **b** scenario where there was no CAP law and the child did (INJURY) or did not (NO INJURY) sustain an injury. **c** scenario where the child sustained an injury in the absence (NO LAW) or presence (LAW) of a CAP law. **d** scenario where there was a CAP law present and the child did (INJURY) or did not (NO INJURY) sustain an injury. **e** scenario for the most disparate conditions, i.e. absence of a CAP law and no injury to the child (NO LAW + NO INJURY) versus presence of a CAP law and injury to the child (LAW + INJURY). Asterisks (*) indicate statistically significant differences for pairwise comparisons: *, *p* < 0.05; **, *p* < 0.01

Scenario 2 involved a child who had accessed a loaded gun that was unsecured in the home under conditions where there was or was not a state CAP law and where the child did or did not sustain an injury. The vast majority of social worker respondents considered the scenario child neglect for all ages included in the study (4–14 years). In the absence of an injury (Fig. 2a), there were no differences in the age curves between scenarios with and without a CAP law. Similarly, in the absence of a law (Fig. 2b) there were no differences in the age curves between scenarios with and without an injury. If the child sustained an injury in the scenario (Fig. 2c), a significantly greater percentage considered the situation child neglect when a CAP law was also present as compared to when it was not for 14 year olds. With a CAP law (Fig. 2d), significantly greater proportions determined the situation child neglect when an injury had also been sustained as compared to when there was no injury for all ages. Figure 2e shows the most disparate situations in the scenario, no law/no injury versus law/injury. In this case, significant differences were noted for ages 6 through 14 years.

## Discussion

We found a strong consensus among social workers who work with children that allowing access or potential access to loaded firearms by children in the home constitutes child neglect. However, one of the factors that affected social worker determinations was the presence of a state CAP law. This was especially true in the scenario where the child had potential access to a loaded firearm; a finding that was previously noted in the determination of child neglect by surveyed child abuse and neglect experts (Evans et al., 2017a). In addition, social workers were significantly more likely to make the determination of child neglect after a child accessed a firearm when they suffered an injury as compared to when they had not if there was also a CAP law in place. Furthermore, and similar to what was found with child abuse and neglect experts (Evans et al., 2017a), almost all social worker respondents affirmed that there should be CAP laws requiring firearms to be safely stored, including separately stored ammunition, to prevent unwanted access to a loaded firearm by children up through 15 years of age.

The majority of studies have found CAP laws to be associated with decreased rates of suicides and unintentional firearm injuries and deaths among children (Hamilton et al., 2018a; Santaella-Tenorio et al., 2016; Cummings et al., 1997; Webster et al., 2004; DeSimone et al., 2013; Webster & Starnes, 2000; Lee et al., 2013; Hepburn et al., 2006). A case-control study showed that youth in homes with safer firearm storage had lower risks of both self-inflicted and unintentional firearm-related injuries (Grossman et al., 2005). Moreover, an analysis of the national Healthcare Cost and Utilization Project-Kids’ Inpatient Database demonstrated strong CAP laws that imposed criminal liability for negligently stored firearms were associated with a 54% reduction in suicidal firearm-related injuries and a 44% reduction in unintentional injuries in children under 18 years as compared with states with no CAP law (Hamilton et al., 2018a). States with weak CAP laws showed no improvement over states with no CAP laws in the study. In addition, states with both CAP laws and stricter firearm legislation have been found to have lower rates of firearm ownership in households with young children, as well as safer storage of firearms in homes that do (Prickett et al., 2014). Encouraging safe storage, including the passage of strong CAP laws, may be as or even more effective than controlling sales in decreasing the mortality and morbidity risks of firearms in the home (Hamilton et al., 2018b).

The vast majority of social workers in the study determined a case as being child neglect when a youth had obtained an unsecured, loaded firearm in the home. The percentages of survey respondents finding child neglect decreased as child age increased in the scenario, mostly when children were 12 years of age and older. However, even when the child was 14 years of age, at least 82% of the social workers believed the circumstance as being child neglect. In a previous survey, child abuse and neglect experts had even higher percentages determining child neglect when evaluating identical scenarios of a child accessing an unlocked and loaded weapon in the home from that seen with the social workers in this study (Evans et al., 2017a).

In reality, parents often fail to take proper measures to ensure children are unable to come into the possession of household firearms. Of homes with children 17 years old and younger, 14–30% have loaded firearms (Schuster et al., 2000; Azrael et al., 2000; Hemenway et al., 1995; Weil & Hemenway, 1992). Among firearm-owning households with children, 43% have at least one firearm unlocked (Schuster et al., 2000), and 6–21% have at least one firearm both unlocked and loaded (Schuster et al., 2000; Stennies et al., 1999; Azrael et al., 2000; Hemenway et al., 1995). Most parents assume their children would leave a firearm alone or tell an adult if they came across one (Connor & Wesolowski, 2003; Farah et al., 1999), and many believe their child could be “trusted” with a loaded firearm (Farah et al., 1999).

However, these beliefs reveal a misconception of child and adolescent development, particularly regarding their curiosity and poor impulse control. A number of studies have demonstrated that most children, and especially males, will handle a weapon they encounter when not under direct adult supervision (Hardy, 2002; Hardy et al., 1996; Jackman et al., 2001). Nearly three-quarters of children 5–14 years of age in homes with firearms know where they are kept, and 36% have actually handled the weapon—contrary to their parents’ expectations (Baxley & Miller, 2006). Even three-year-olds can generate the pressure necessary to pull the trigger of 92.5% of the handguns in circulation (Naureckas et al., 1995). Although nearly all firearm owners believe that talking to youth about gun safety is essential (Parker et al., 2017), safety instruction does not alter the probability of a child handling a found firearm (Hardy, 2002; Hardy et al., 1996; Jackman et al., 2001).

The high prevalence of unsecure firearm storage and the discordance between parent’s perceptions and how children actually behave reveal attitudes that are significant obstacles to achieving universal firearm safety in households with children. One way to potentially modify these attitudes and change behavior is to make parents and the public mindful of what experts believe constitutes child neglect with regards to firearms. More importantly, parents and other caregivers becoming cognizant that child protective services are investigating and finding child neglect when firearms are not properly secured from children may be a more effective deterrent.

The vast majority of surveyed social workers also found the situation child neglect when 4–14 year olds had potential access to an unlocked and loaded firearm, but the proportions significantly increased for all ages when a CAP law was present. In addition, more than half of social workers that did not regard potential access to a loaded firearm as child neglect at any age in the absence of a law reversed that decision if a CAP law was included in the scenario. Child neglect determinations should be based on risk assessment, and the risks are the same regardless of whether there’s a CAP law. However, our results support the hypothesis that CAP laws make a difference not only in their potential direct effects on reducing pediatric firearm-related injuries and deaths, but also in how they may impact social worker’s determinations of child neglect.

### Limitations

Since only NASW members whose practice was child welfare were surveyed and our response rate was relatively low, the generalizability of the study may be limited. It is also possible that selection bias may have resulted from incomplete membership participation, and there were some differences in demographics between those that participated in the survey and non-respondents. However, the study results are very similar to that found with another group of experts in child neglect (Evans et al., 2017b). In addition, the participant’s determination of child neglect was after consideration of a survey scenario; it is uncertain if respondent’s actions would be similar or vary when evaluating actual cases. Despite limitations, this study addresses a significant knowledge gap as it is the first to assess social workers’ opinions regarding firearm access and child neglect.

## Conclusions

Of child/family welfare social workers who responded to our study, the vast majority determined that scenarios of children’s potential and actual access of unsecured and loaded firearms represented child neglect. However, these determinations of neglect were affected in some cases by the presence of CAP laws. Additionally, the preponderance of social workers in the study strongly agreed that there should be strict CAP laws, even through 15 years of age. As CAP laws markedly vary between states and many have none, determinations of child neglect when children access or have potential access to firearms are likely inconsistent from state to state. This might be improved with passage of strict universal CAP laws. Additionally, parents and other caretakers being held accountable for child neglect when there is access or potential access to firearms in the home might provide a strong stimulus for properly storing firearms. Reducing childhood firearm access could in turn decrease intentional (including suicide) and unintentional firearm-related deaths and injuries.

## Abbreviations

AAP: American Academy of Pediatrics
BSW: Bachelors of Social Work
CAP: child access prevention
DSW: Doctorate of Social Work
MSW: Masters of Social Work
NASW: National Association of Social Workers
RUCA: Rural Urban Commuting Area
SOCAN: Section on Child Abuse and Neglect
SPSS: IBM Statistics Package for the Social Sciences
